# Lysyl oxidase (LOX) limits VSMC proliferation and neointimal thickening through its extracellular enzymatic activity

**DOI:** 10.1038/s41598-018-31312-w

**Published:** 2018-09-05

**Authors:** Saray Varona, Mar Orriols, María Galán, Anna Guadall, Laia Cañes, Silvia Aguiló, Marc Sirvent, José Martínez-González, Cristina Rodríguez

**Affiliations:** 1 Instituto de Investigaciones Biomédicas de Barcelona (IIBB-CSIC), Barcelona, Spain; 2 CIBER de Enfermedades Cardiovasculares (CIBERCV), ISCIII, Madrid, Spain; 3 Instituto de Investigación Biomédica Sant Pau (IIB-Sant Pau), Barcelona, Spain; 4 Institut de Recerca del Hospital de la Santa Creu i Sant Pau-Programa ICCC, Barcelona, Spain; 5 Hospital Universitari Germans Trias i Pujol, Badalona, Spain

## Abstract

Lysyl oxidase (LOX) plays a critical role in extracellular matrix maturation and limits VSMC proliferation and vascular remodeling. We have investigated whether this anti-proliferative effect relies on the extracellular catalytically active LOX or on its biologically active propeptide (LOX-PP). High expression levels of both LOX and LOX-PP were detected in the vascular wall from transgenic mice over-expressing the full-length human LOX cDNA under the control of SM22α promoter (TgLOX), which targets the transgene to VSMC without affecting the expression of mouse LOX isoenzymes. TgLOX VSMC also secrete high amounts of both mature LOX and LOX-PP. Wild-type (WT) mouse VSMC exposed to VSMC supernatants from transgenic animals showed reduced proliferative rates (low [^3^H]-thymidine uptake and expression of PCNA) than those incubated with conditioned media from WT cells, effect that was abrogated by β-aminopropionitrile (BAPN), an inhibitor of LOX activity. Lentiviral over-expression of LOX, but not LOX-PP, decreased human VSMC proliferation, effect that was also prevented by BAPN. LOX transgenesis neither impacted local nor systemic inflammatory response induced by carotid artery ligation. Interestingly, in this model, BAPN normalized the reduced neointimal thickening observed in TgLOX mice. Therefore, extracellular enzymatically active LOX is required to limit both VSMC proliferation and vascular remodeling.

## Introduction

Vascular remodeling is a hallmark of cardiovascular diseases such as atherosclerosis and restenosis. Vascular remodeling results from an abnormal response to injury which involves both the dedifferentiation of vascular smooth muscle cells (VSMC) to a synthetic and proliferative phenotype and a substantial extracellular matrix (ECM) reorganization^1–3^. Environmental cues, including those related with an altered ECM composition and structure, affect VSMC proliferation and play a pivotal pathophysiologic role in the development of intimal hyperplasia^4,5^. Therefore, further research on ECM-dependent regulatory mechanisms governing VSMC proliferation will contribute to identify more effective therapeutic approaches for vascular diseases.

The covalent cross-link of collagen and elastin fibers is a fundamental process in ECM scaffolding. This process, catalyzed by lysyl oxidase (LOX), occurs via the oxidative deamination of lysine and hydroxylysine residues yielding highly reactive peptidyl semialdehydes that spontaneously condense to form the intra- and intermolecular covalent cross-linkages responsible for ECM stability^6–8^. LOX is synthesized as a prepro-enzyme that undergoes a series of post-translational modifications in endoplasmic reticulum and Golgi to yield a 50 kDa pro-enzyme. This precursor is secreted to the extracellular space, where it is proteolized to release the catalytically active LOX form (32 kDa) and its propeptide (LOX-PP). Unexpectedly, intracellular active forms of LOX have been identified in different cell types, including VSMC, exerting intracellular functions^6,9,10^. Furthermore, several LOX-dependent biological responses including tumor suppression, inhibition of basic fibroblast growth factor (bFGF) signaling, suppression of neuronal development and induction of cell differentiation are not dependent on LOX catalytic activity but have been ascribed to LOX-PP^11–14^.

Our previous research supports the contribution of LOX to the pathophysiology of atherosclerosis, restenosis and hypertension^15–20^. Using a transgenic mouse model that over-expresses the full-length human LOX cDNA in VSMC (TgLOX) we have recently reported that LOX attenuates vascular remodelling induced by carotid artery ligation by limiting VSMC proliferation^20^. In VSMC in culture Hurtado *et al*. evidenced that treatment with purified LOX-PP reduced proliferative rates^21^. However, high concentrations of the propeptide were used in these assays and no *in vivo* studies have been performed to clarify this function of LOX-PP. Therefore, it is still unclear whether the vascular anti-proliferative activity of LOX relies on the extracellular catalytically active form of this enzyme or is dependent on the biological activity of either intracellular forms or the propeptide. In this work, we sought to investigate the specific LOX form underlying LOX-mediated inhibition of VSMC proliferation.

## Results

### VSMC supernatants from TgLOX mice limit cell proliferation

**Figure 1:**
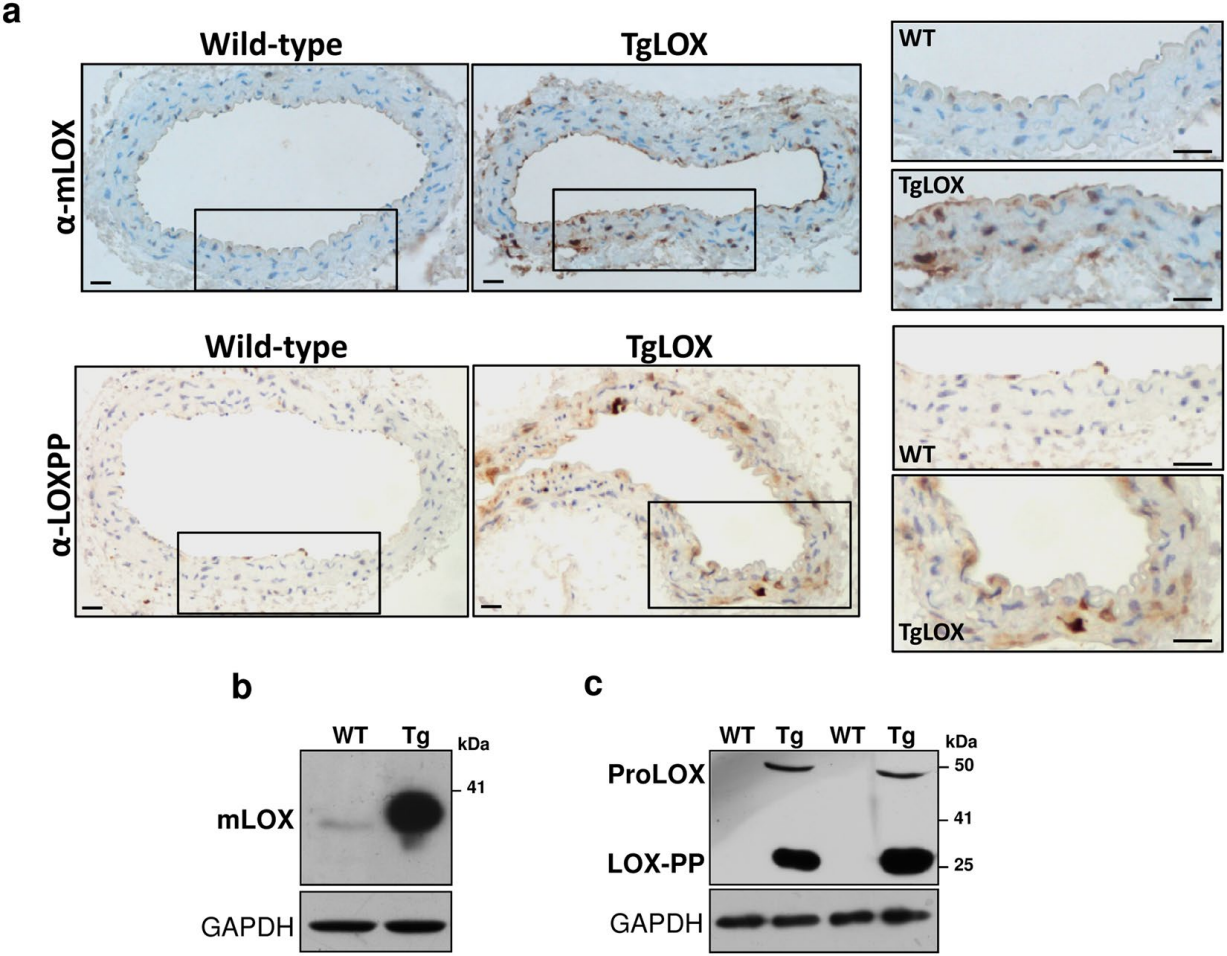
Mature LOX and LOX-PP were over-expressed in the vascular wall and in VSMC from TgLOX mice. (**a**) Representative immunohistochemical analysis showing LOX and LOX-PP staining (brown color) in aorta from both wild-type and TgLOX mice. The indicated areas are shown at high magnification (bars = 20 µm). (**b**,**c**) Mature LOX and LOX-PP protein levels were determined by Western-blot in VSMC supernatants from these animals. The position of the pro-enzyme (ProLOX), mature LOX (mLOX) and LOX-PP forms are indicated. GAPDH was analyzed as loading control. Representative immunoblots from 3 independent experiments were shown. WT: wild-type; Tg: TgLOX. Displayed blots are not cropped from different gels or different parts of the same gel and images conform the digital image and integrity policies of the journal.

Immunohistochemical analysis of LOX expression using an antibody that recognizes a region of the catalytic domain demonstrated the over-expression of human LOX in aortic media from TgLOX mice. Similarly, an increased staining was detected in the vascular wall from transgenic mice using an antibody raised against LOX-PP that recognizes both the pro-enzyme of LOX and its propeptide (Fig. 1a). Accordingly, high levels of mature LOX (Fig. 1b) as well as LOX-PP and proLOX (Fig. 1c) were detected in VSMC supernatants from TgLOX mice. LOX-PP migrates as a 35 KDa protein under reducing conditions due to multiple glycosylations and its highly basic disordered nature, consistent with published reports^13^.

**Figure 2:**
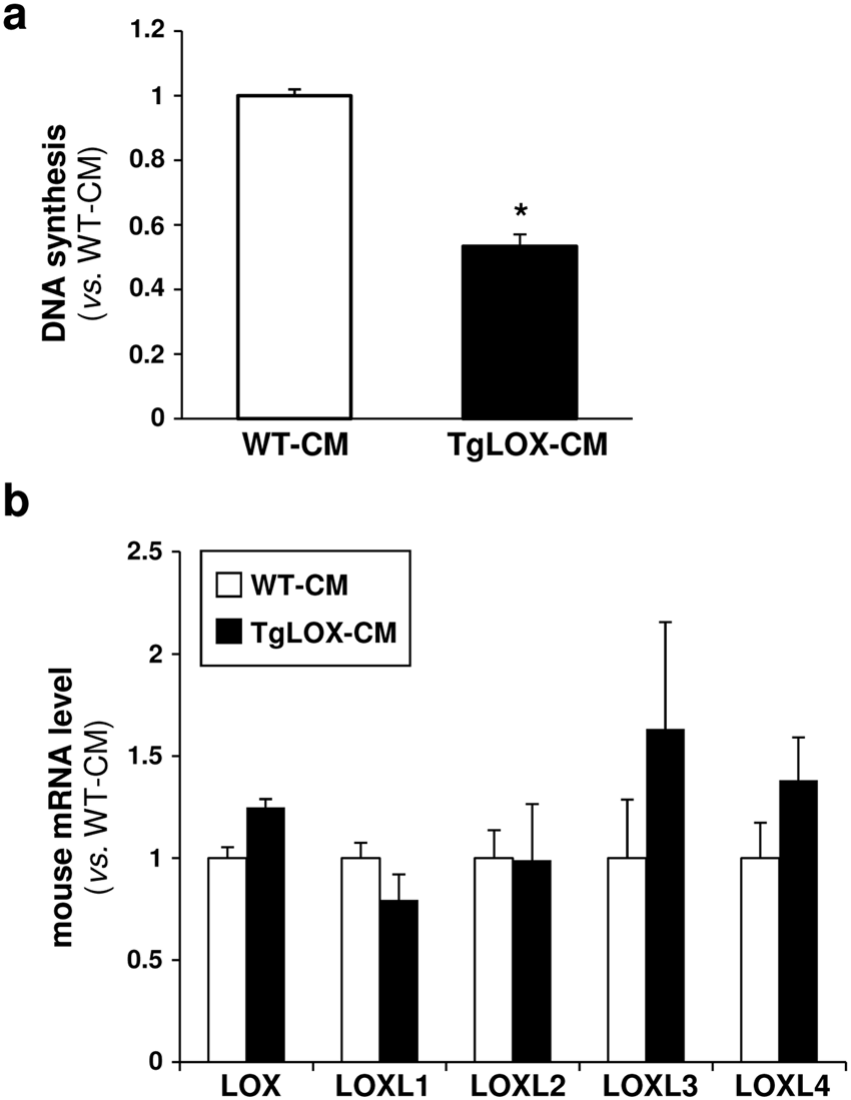
Extracellular forms are involved in the attenuated cell proliferation induced by LOX transgenesis. (**a**) Aortic VSMC isolated from wild-type (WT) mice were serum-starved for 24 h and then exposed to conditioned media from transgenic (TgLOX-CM; black bars) or WT VSMC (WT-CM; white bars). Cell proliferation was analyzed by the [^3^H]-thymidine incorporation method. Results are represented as mean ± SD. *P < 0.002 *vs*. WT-CM stimulated cells (Mann–Whitney test; n = 6). (**b**) Endogenous LOX and LOXLs mRNA levels were determined in these cells by real-time PCR. Data are expressed as mean ± SD (Mann–Whitney test; n = 4).

VSMC over-expressing LOX displayed lower proliferative rates than control cells (see Supplementary Fig. S1), in agreement with previous results^20^. To determine whether extracellular LOX forms could be involved in this effect, cells from wild-type mice were exposed to conditioned media from TgLOX cells. Mouse VSMC cultured in supernatants from transgenic cells showed lower [^3^H]-thymidine incorporation rates than those growing in conditioned media from normal cells (Fig. 2a). The reduction in cell proliferation (aprox. 53%) was similar to that detected in TgLOX VSMC cultured under conventional conditions^20^. LOX transgenesis did not alter the endogenous expression of LOX or LOX-like isoenzymes (Fig. 2b).

### BAPN prevented the decrease in VSMC proliferation evoked by LOX

**Figure 3:**
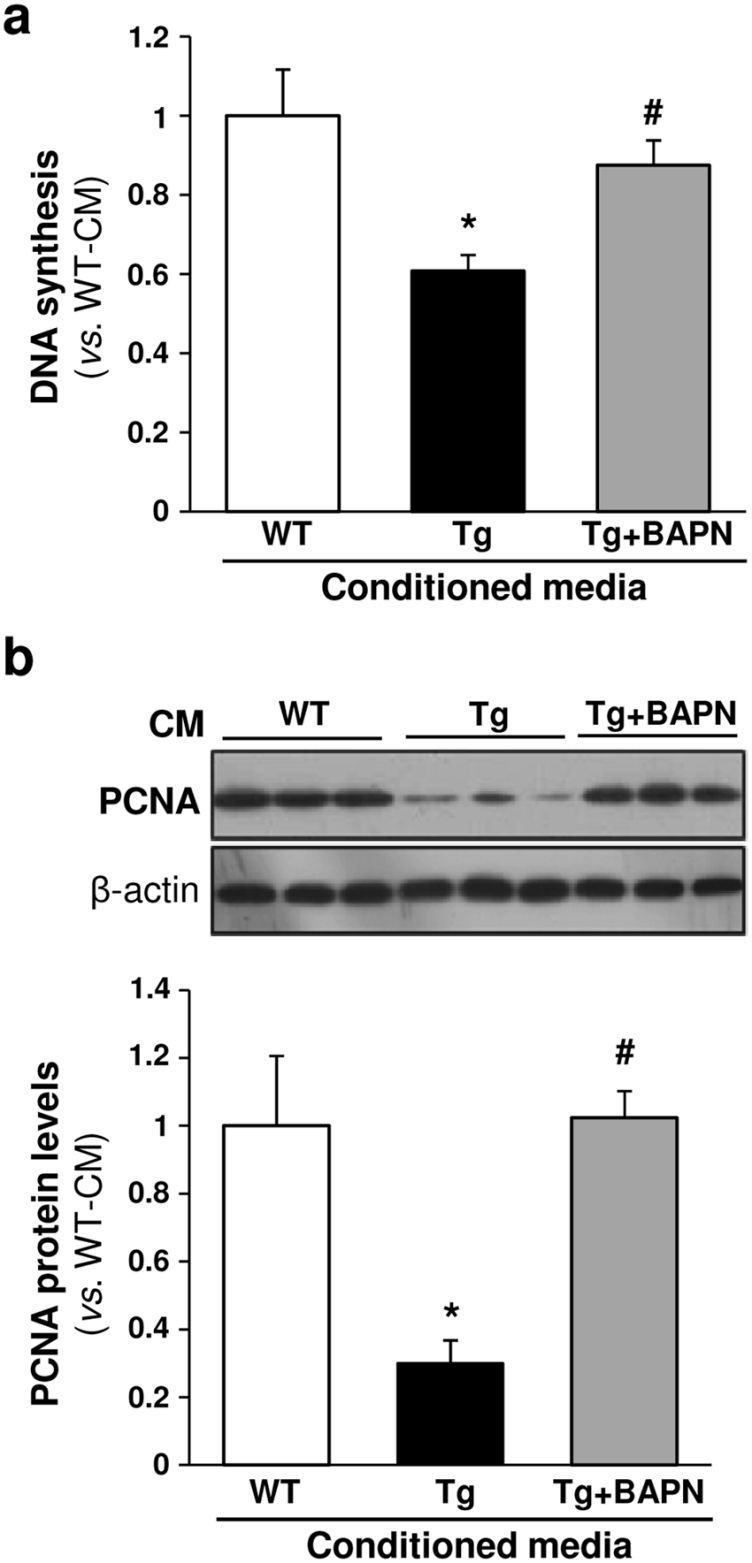
BAPN prevents the reduction in VSMC proliferation induced by LOX transgenesis. Aortic VSMC isolated from wild-type (WT) mice were serum-starved for 24 h and then exposed to conditioned media from transgenic VSMC (Tg; black bars) stimulated or not with BAPN (Tg + BAPN; grey bars) or from WT VSMC (white bars). (**a**) Cell proliferation was analyzed by the [^3^H]-thymidine incorporation method. Results are represented as mean ± SD. *P* < 0.0001: * *vs*. cells stimulated with conditioned media from WT VSMC; ^#^ *vs*. cells stimulated with conditioned media from Tg VSMC (One-way ANOVA; at least n = 6). (**b**) PCNA protein levels were assessed by Western-blot (upper panel). The bars graph (lower panel) shows the values, obtained by densitometric analysis of Western-blots. Results normalized by β-actin levels are shown as mean ± SD (n = 4; *P* < 0.05: * vs. cells stimulated with conditioned media from WT VSMC; ^#^ *vs*. cells stimulated with conditioned media from Tg VSMC; Kruskal-Wallis). Displayed blots are not cropped from different gels or different parts of the same gel and images conform the digital image and integrity policies of the journal.

To determine the contribution of LOX catalytic activity to the regulation of VSMC proliferation, we analyzed whether β-aminopropionitrile (BAPN), an irreversible and specific inhibitor of LOX activity, influences proliferative rates. Wild-type VSMC were exposed to conditioned media derived from TgLOX cells supplemented with or without BAPN. We observed that BAPN attenuated the decrease in [^3^H]-thymidine incorporation observed in cells exposed to VSMC supernatants containing high extracellular levels of LOX (Fig. 3a) and also normalized proliferating cell nuclear antigen (PCNA) levels (Fig. 3b). BAPN neither affected the endogenous mRNA levels of LOX or LOXL isoenzymes (see Supplementary Fig. S2) nor endogenous LOX protein levels (data not shown). Therefore, our data support the involvement of LOX enzymatic activity in the reduced VSMC proliferation triggered by this enzyme.

### The negative regulation of VSMC proliferation was independent of LOX-PP

**Figure 4:**
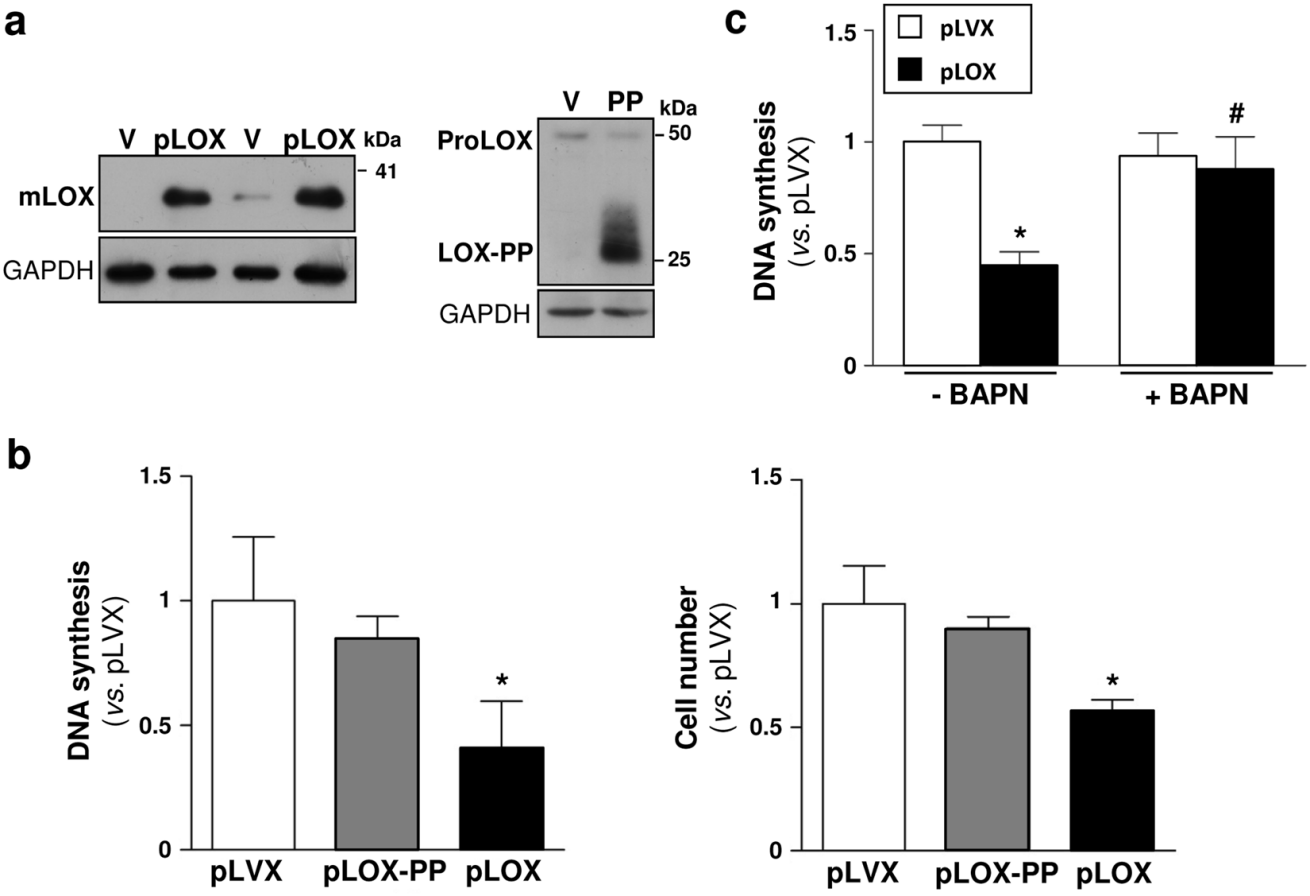
Over-expression of LOX-PP did not affect VSMC proliferation. Human VSMC were transduced with a lentiviral vector encoding for full-length LOX (pLOX; black bars), LOX-PP (pLOX-PP; grey bars) or with the corresponding empty vector (pLVX; V; white bars). (**a**) Immunoblots corresponding to mature LOX (mLOX; left panel) and LOX-PP (right panel) are shown. The position of the pro-enzyme (ProLOX, right panel), detected with the antibody against the propeptide, was also indicated. GAPDH was analyzed as a loading control. Displayed blots are not cropped from different gels or different parts of the same gel and images conform the digital image and integrity policies of the journal. Representative immunoblots from 3 independent experiments were shown. (**b**) Transduced VSMC were serum-starved for 24 h and then stimulated with 20% FCS. Cell proliferation was evaluated by the [^3^H]-thymidine incorporation method (left panel) or by cell count (right panel) in these cells. Results represented as mean ± SD. *P < 0.003 *vs*. pLVX (Kruskal-Wallis; at least n = 6). (**c**) [^3^H]-thymidine incorporation into DNA assessed in human VSMC transduced with lentiviral particles for full-length LOX (pLOX; black bars), or the corresponding empty vector (pLVX; white bars) treated or not with BAPN (500 µM). Results are represented as mean ± SD. *P* < 0.0001: * *vs*. pLVX; ^#^ *vs*. pLOX transduced cells (Two-way ANOVA; at least n = 9).

To further characterize the mechanisms underlying the LOX-mediated anti-proliferative activity, either full-length LOX or LOX-PP were over-expressed in human VSMC by lentiviral transduction (see Supplementary Fig. S3). Western blot assays in VSMC supernatants evidenced that both LOX and LOX-PP transduction led to an enhanced secretion of mature LOX and LOX propeptide, respectively (Fig. 4a). While LOX-transduced cells exhibited a significant decrease in [^3^H]-thymidine incorporation and cell number (Fig. 4b), the over-expression of LOX-PP in these cells did not alter proliferative rates (Fig. 4b). Interestingly, BAPN prevented the reduction in [^3^H]-thymidine incorporation rates induced by LOX transduction, further supporting the involvement of LOX catalytic activity in the anti-proliferative properties of this enzyme in VSMC (Fig. 4c).

### BAPN ameliorated the attenuated vascular remodeling exhibited by TgLOX mice

**Figure 5:**
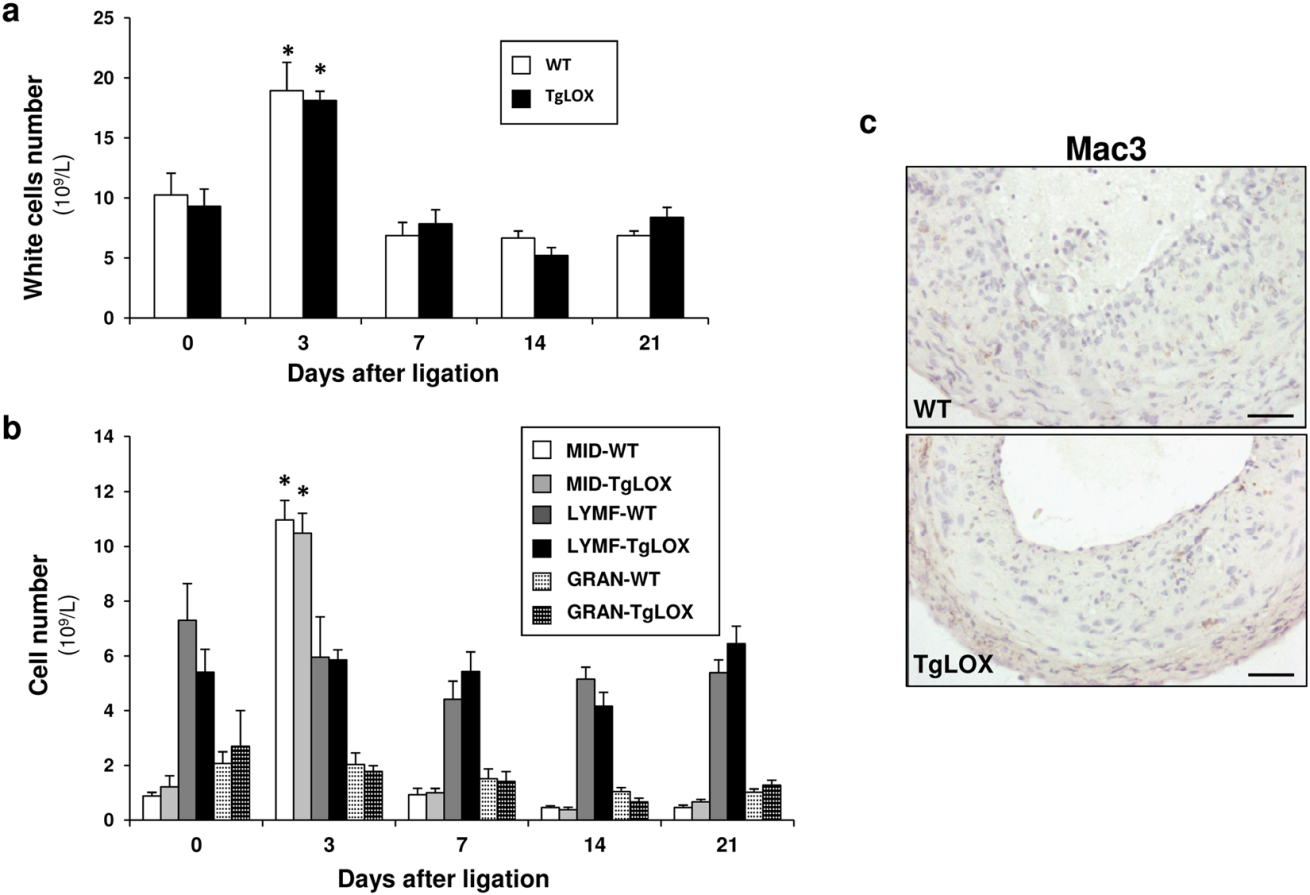
LOX transgenesis did not affect the systemic and local inflammatory response after carotid artery ligation. (**a**) Number of circulating white cells in wild-type (WT) and TgLOX mice after carotid artery ligation. (**b**) Count of the number of circulating monocytes (MID: Mid-sized cells; a subgroup of white blood cells that consists primarily of monocytes), lymphocytes (LYMF) and granulocytes (GRAN) in these animals. Data are expressed as mean ± SEM (*p < 0.001 vs. WT or TgLOX mice at time 0; Two-way ANOVA; n = 6). (**c**) Mac3 immunostaining in hematoxylin counterstained cross-sections of ligated carotid arteries from wild-type and TgLOX mice 3 weeks after ligation (n = 3). Bar: 20 µm.

We have previously reported that LOX transgenesis attenuates vascular remodeling induced by carotid artery ligation^20^. To further characterize the mechanisms involved in the reduced vascular remodeling in TgLOX mice, we investigated whether the inflammatory response triggered by carotid artery ligation could be altered by vascular LOX over-expression. However, LOX transgenesis did not entail differences in the transient vascular inflammatory response induced by carotid artery ligation. The analysis of the number of circulating white-blood cells throughout the three weeks of the study revealed a similar transient increase in monocytes 3 days after ligation in both wild-type (WT) and TgLOX mice (Fig. 5a,b). Furthermore, vascular macrophage infiltration in ligated carotid arteries was analyzed by Mac3 immunostaining. Low macrophage infiltration in the intima was detected and no differences between wild-type and TgLOX animals were observed (Fig. 5c).

**Figure 6:**
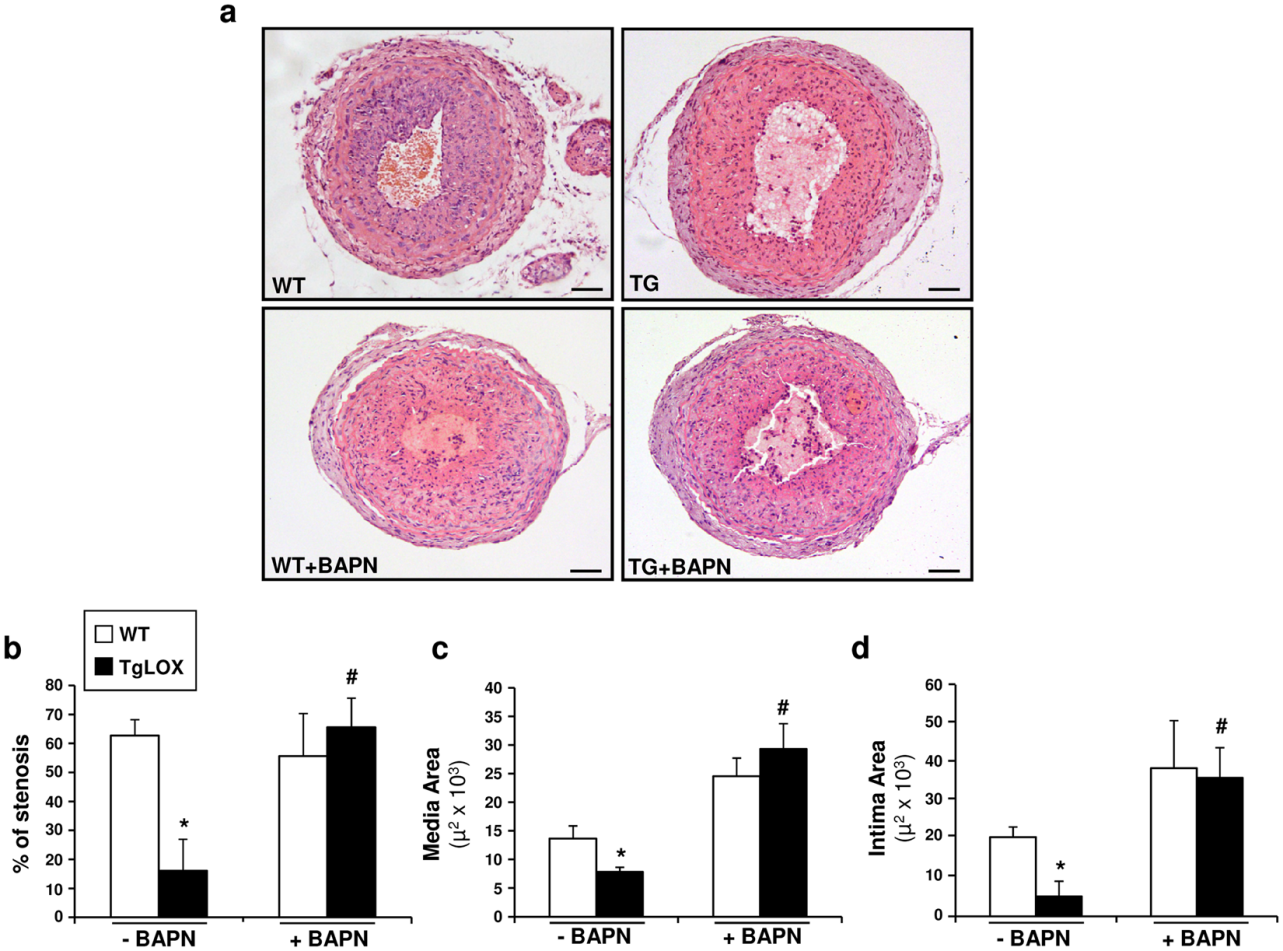
BAPN ameliorates the reduced vascular remodeling after carotid artery ligation. Left common carotid arteries were harvested from TgLOX mice and wild-type (WT) littermates treated or not with BAPN 21 days after permanent ligation. Morphometric analysis was performed as described in the Material and Methods section. (**a**) Representative microphotographs of injured carotid arteries stained with hematoxylin and eosin in these animals. Bar: 100 µm. (**b**) Analysis of the percentage of stenosis and intima and media areas from sections covering the region 1.4 to 1.6 mm from the ligation site. Three cross-sections for each carotid artery were measured, and the data were averaged. Data are expressed as mean ± SEM. *P* < 0.05: * *vs*. wild-type animals; ^#^ *vs*. TgLOX mice (Two-way ANOVA; at least n = 6).

As described above, our *in vitro* data support the role of LOX catalytic activity in the control of VSMC proliferation. Therefore, we aimed to determine whether inhibition of LOX activity by BAPN could influence the reduced neointimal thickening observed in TgLOX mice using the carotid artery ligation model. BAPN was administered to both wild-type and TgLOX mice at a dose of 100 mg/kg/day, chosen on the basis of previous reports indicating LOX inhibition without evident side-effects^22–26^. As shown in Fig. 6, BAPN partially prevented the decrease in neointimal growth detected in TgLOX mice, limiting the reduction in the percentage of stenosis and in the intima and media areas.

## Discussion

LOX is a multifunctional enzyme that, besides its well-known involvement in ECM scaffolding, participates in multiple biological responses, including the regulation of cell transformation, migration and proliferation in different cells and tissues^6–8,27^. We have previously shown the critical contribution of LOX to the control of endothelial homeostasis^16–19^ and to the regulation of vascular remodeling by modulating cell proliferation, ECM calcification and ROS production^15,20,28^. Neointimal thickening induced by carotid artery injury was attenuated in a transgenic mouse model that specifically over-expresses LOX in VSMC, consistent with the lower proliferative rates exhibited by VSMC from these animals^20^. Considering the complex biology of this enzyme, however, previous studies have not dealt with the specific mechanisms involved in the anti-proliferative activity of LOX in VSMC. Here, we have evidenced that LOX limits VSMC proliferation and neointimal thickening after arterial wall injury through its extracellular enzymatic activity.

TgLOX mice show an intense expression of LOX in the vascular wall as we previously described^20^. The immunohistochemical analysis using an antibody raised against an internal region of LOX-PP, which also detects the pro-enzyme, evidenced the increased vascular levels of these forms and depicted a significant extracellular location. Furthermore, immunoblotting showed that besides the catalytically active form of LOX, VSMC from TgLOX mice secrete high amounts of LOX-PP, evidencing that the product of the transgene was properly expressed and processed.

We and others documented the anti-proliferative properties of LOX in VSMC^20,21^, however, it is unclear whether this effect requires LOX catalytic activity, depends on LOX-PP or even it could be mediated by intracellular LOX forms. In this regard, while in sarcoma cells opposite effects on cell proliferation have been ascribed to the catalytic domain of LOX and LOX-PP^29^, in prostate cancer, active LOX seems to exhibit a dual behavior acting either as a tumor-suppressor or as a tumor-promoter probably depending on the stage of disease^30^. Concerning intracellular LOX-dependent mediated responses, enzymatically active LOX has been identified in nuclei from VSMC and it has been suggested that this nuclear form could modulate cell proliferation through a catalytic activity-dependent limitation of nuclear mass^31^. Here, we have determined that conditioned media from TgLOX VSMC, which contains high amounts of both mature LOX and LOX-PP, reduced proliferation to rates largely resembling those previously reported for VSMC over-expressing LOX^20^. Therefore, LOX transgenesis appears to act primarily in a paracrine manner to inhibit VSMC growth.

Interestingly and in contrast to previous studies^21^, our results exclude the involvement of LOX-PP in the control of VSMC growth. LOX-PP lentiviral-transduction in human VSMC did not affect proliferative rates, while the over-expression of the entire LOX pro-enzyme, which was properly processed, significantly reduced cell growth. Conversely, Hurtado *et al*. reported that exogenously added LOX-PP inhibited proliferation of rat VSMC. However, this effect required the use of high concentrations of the recombinant propeptide and therefore its physiological significance remains doubtful^21^. Further, and unlike Hurtado *et al*., we did not use tag epitopes, but expressed the human full-length propeptide including the native LOX signal peptide, which directs to the secretory pathway, assuring the proper subcellular location and biological activity of the ectopically over-expressed LOX-PP. This could underlie the observed discrepancies with regard to the role of LOX-PP in the control of VSMC growth.

It has been previously described that vascular LOX over-expression reduces MCP-1 secretion and limits vascular macrophage infiltration in a model of abdominal aortic aneurysm^32^. In agreement, we reported a LOX-dependent decrease in vascular MCP-1 expression in TgLOX mice^20^; however, LOX over-expression had neither consequence on the recruitment of inflammatory cells in the carotid artery nor on the characteristic systemic inflammatory response transiently induced by this procedure^33,34^. Therefore, in the carotid artery ligation model, local and systemic inflammatory responses did not differ between wild-type and TgLOX mice excluding the imbalance in the inflammatory response as a cause for the LOX-dependent attenuation of vascular remodeling.

Likewise, we established that the anti-proliferative effect induced by LOX requires LOX enzymatic activity since it was inhibited by BAPN. BAPN is a specific and irreversible inhibitor of LOX activity that acts as a suicide substrate^35^ and constitutes a fundamental tool to determine whether LOX biological activities rely on its catalytic function^22–26^. Although BAPN could inhibit other lysyl oxidase isoenzymes, LOX accounts for more than 80% of lysyl oxidase activity in VSMC^36^. Moreover, the endogenous expression of LOX and LOXL isoenzymes was unaltered by the exposure of VSMC to either conditioned media from TgLOX cells or BAPN; therefore the effect of BAPN in these cells would be due to the inhibition of the over-expressed LOX rather than by the blockade of LOXLs. Consequently, these data support that secreted and enzymatically active LOX is responsible for growth inhibition in VSMC. It should be noted, that research in animal models of arterial injury have yielded discordant results regarding the effect of BAPN in vascular remodelling and the potential pathological role of LOX^37,38^. This issue remained unclear until the development of TgLOX mice, animals that exhibit an attenuated vascular remodeling in response to injury. Our *in vivo* studies evidences that BAPN significantly prevented the reduction in neointimal thickening induced by carotid artery ligation in TgLOX mice, further supporting that active LOX is required to limit vascular remodeling.

Nevertheless, it remains to be elucidated whether this LOX catalytic-dependent effect lies in its primary action on ECM organization or rather in other mechanisms. It should be noted, that in VSMC in culture long-term BAPN treatment should be necessary to significantly reduce the deposition of mature collagen, as we have recently observed^28^. In fact, no changes were observed in collagen I deposition analyzed by confocal analysis in VSMCs from TgLOX mice exposed for 24 h to BAPN (data not shown). Therefore, at least in VSMC, the alteration of ECM structure does not seem to be the responsible for the LOX-dependent inhibition of cell proliferation. This could be different *in vivo*, however, due to the active ECM remodeling triggered by the ligation of the carotid artery, and because the daily administration of BAPN for 21 days could affect ECM structure and/or stability. Further, LOX is able to oxidatively modify other proteins relevant in cell chemotaxis and mitogenesis such as PDGF receptor and bFGF, altering cell behaviour^39,40^. Hence, considering the complex biology of LOX, further research is required to clarify this issue.

In summary, our results evidence that the extracellular enzymatic activity of LOX drives paracrine anti-proliferative effects on VSMC. Further, our results rule out a role for the biologically active LOX propeptide and seem to discard an autocrine regulation by intracellular forms of this enzyme in such effect. These results underscore the importance of targeting LOX activity to modulate vascular remodeling.

## Materials and Methods

### Animal handling

Studies were performed in transgenic mice that exhibit a SMC–specific expression of human LOX (TgLOX) under a C57BL/6 J background^15,20,28^ and in their non-transgenic littermates (wild-type; WT). Animals were bred in the Animal Experimentation Unit of the Institut Català de Ciències Cardiovasculars (ICCC, Barcelona, Spain) and housed in a controlled, specific pathogen-free environment. All animal handling procedures were performed in compliance with the principles and guidelines established by the American Physiological Society for animal research, and all procedures were reviewed and approved by the Ethical Committee at the ICCC as stated in Law 5/1995, 21 June, passed by the Generalitat de Catalunya. Animals were taken care of and used according to the Spanish Policy for Animal Protection RD53/2013, which meets the European Union Directive 2010/63/UE on the protection of animals used for experimental and other scientific purposes.

### Carotid artery ligation experiments and morphometric analysis

Permanent ligation of the left common carotid artery was performed as previously described^20,41^. Four-month-old mice were distributed into four groups: WT (n = 12), WT mice receiving BAPN (100 mg/kg/day; ip; n = 7) from the day before surgical intervention and throughout the experimental procedure, TgLOX (n = 10) and TgLOX treated with BAPN (n = 6). Animals were anaesthetized by intraperitoneal injection of a ketamine/medetomidine/buprenorphine cocktail. A midline neck incision was made and the left common carotid artery was carefully dissected under a microscope and ligated with a 7-0 silk suture just proximal to its bifurcation. After 3 weeks under standard chow diet the animals were anaesthetized with ketamine (75 mg/kg) and medetomidine (1 mg/kg) and sacrificed by thoracotomy. Carotid arteries were then excised, fixed in 4% paraformaldehyde/0.1 M PBS (pH 7.4) for 24 h, and embedded in paraffin for morphometric and immunohistochemical analysis^41^. Serial sections (5 µm thick) of paraffin-embedded arteries were cut using a microtome (Leica JUNG RM 2055). Cross-sections at 1.4, 1.5 and 1.6 mm from the ligation site were stained with hematoxylin and eosin. Images of these sections were captured with a microscope (Olympus BX50) at 200X magnification, and analyzed using the image analysis software ImageJ (NIH, Bethesda, MD). The circumferences of the lumen, internal elastic lamina (IEL) and external elastic lamina (EEL) were obtained by tracing their contours on digitalized images. Medial, intimal and lumen areas were calculated from these parameters. The media area is the area between EEL and IEL, and the intima area was calculated by subtracting the lumen area from the IEL area. The % stenosis was calculated as: [intima area/(intima area + lumen area)] × 100^20,41^. Measures corresponding to the three sections for each carotid artery were averaged.

### Immunohistochemistry

Mouse aortas were fixed overnight in 4% paraformaldehyde/0.1 M PBS (pH 7.4), embedded in paraffin, and sectioned into 5 mm sections. Deparaffinized sections were rehydrated, subjected to antigen retrieval (10 mM citrate pH 6.0) and blocked with 10% serum/PBS. Antibodies against LOX (ab31238, Abcam), LOX-PP (NB110-41568, Novus Biologicals) and MAC3 (sc-19991, Santa Cruz Biotechnology Inc., Europe) were used. Sections were incubated with the corresponding biotinylated secondary antibodies. Immunocomplexes were detected after incubation with Vectastain Elite ABC reagent (PK6100; Vector Laboratories, Burlingame, CA, USA) and 3,3′-diaminobenzidine substrate.

### Peripheral Blood Analysis

Peripheral blood samples were drawn from TgLOX and wild-type mice by tail snip at baseline and 3, 7, 14 and 21 days after carotid artery ligation. The number of total white blood cells and that of monocytes, granulocytes and lymphocytes was analyzed within 10 minutes using an automated counter (Medonic CA-620, Medicon eG).

### Cell culture

Aortic VSMC from mouse were obtained by the explant technique as previously described^41^. Briefly, endothelium-denuded medial tissue was cut into 1 to 2 mm cubes that were transferred to a 25 cm^2^ culture flask containing 5 mL of pre-warmed DMEM supplemented with 10% fetal calf serum (FCS; Biological Industries) and antibiotics (100 U/mL penicillin and 0.1 mg/mL streptomycin). VSMC migrate out from the explants within 2 to 3 weeks. Then, after removing the explants from the flask surface, cells were trypsinized, used as P1 stage cells, and routinely subcultured. Mouse VSMC were cultured in DMEM (Invitrogen) supplemented with 10% FCS, antibiotics and 2 mM L-glutamine (Invitrogen). Human VSMC were obtained from non-atherosclerotic arteries of hearts removed in transplant surgeries at the Hospital de la Santa Creu i Sant Pau (Barcelona, Spain) and cultured in M199 (Gibco) supplemented with 20% FCS, 2% human serum, 2 mM L-glutamine and antibiotics as previously described^42^. All the procedures were approved by the Reviewer Institutional Committee on Human Research of the Hospital de la Santa Creu i Sant Pau and conforms to the Declaration of Helsinki. No organ/tissues were procured from prisoners. Written informed consent was obtained from each patient. Cells between passages 3 to 6 were seeded in multiwell plates. In some experiments, VSMC from WT mice were incubated with conditioned media from TgLOX cells supplemented or not with BAPN (500 µM), an inhibitor of LOX activity, and the consequences on proliferative rates were determined as described below.

### Lentivirus production and VSMC infection

The human LOX cDNA was obtained by PCR amplification from clone HLO20 and cloned into the pLVX-puro lentiviral vector to generate pLVX/LOX (pLOX) as previously described^20^. The human LOX-PP lentiviral vector was generated following the strategy previously described by Agra *et al.*^29^. The LOX-PP cDNA was amplified by PCR with the following forward and reverse oligonucleotides: 5′-GGGCTCGAGCAATCTGGCAAAAGGAGTGATGC-3′ (forward; *Xho*I site is underlined) and 5′-GGGGGATCCGTCAGAGTACTTGTAGGGGTTGTA-3′ (reverse; *Bam*HI site is underlined). This cDNA was cloned into the *Xho*I and *BamH*I sites of the pLVX empty vector. The empty vector was used as control. These constructs were transfected in HEK 293 T cells according to the Lenti-X^TM^ Lentiviral Expression System (Clontech) in order to obtain viral particles. After 48 h, cell culture supernatants containing recombinant lentiviruses were harvested, centrifuged at 500 × *g* for 5 min at 4 °C and then filtered through 0.45 μm PVDF membrane filters. The viral titer was evaluated by transduction of HeLa cells with 10-fold serial dilutions of the lentivirus stock. Lentiviral particles were added to cells in medium containing Polybrene (8 μg/ml; Sigma-Aldrich), which was changed 24 h later. The consequence of LOX and LOX-PP over-expression by lentiviral infection on VSMC proliferation was evaluated after 10 days of puromycin selection.

### Real-time PCR

Total RNA was isolated from both cultured cells and mouse tissues using Ultraspec^TM^ (Biotecx) or the RNeasy Micro kit (Qiagen), respectively. RNA (1 μg) was reverse transcribed into cDNA using the High Capacity cDNA Reverse Transcription Kit (Applied Biosystems) in the presence of random hexamers. Quantification of mRNA levels was performed by real-time PCR using an ABI PRISM 7900HT sequence detection system (Applied Biosystems) and specific primers and probes provided by the Assay-on-Demand system for hLOX (Hs00184700_m1), murine LOX (Mm00495386_m1), murine LOXL1 (Mm01145738_m1), murine LOXL2 (Mm00804740_m1), murine LOXL3 (Mm01184865_m1), murine LOXL4 (Mm00446385_m1). Forward and reverse primers used for human LOX-PP analysis by real-time PCR with SYBR Green [designed by Primer-Basic Local Alignment Search Tool (BLAST); National Center for Biotechnology Information, Bethesda, MD, USA] are: 5′-GTCAGAGTACTTCTTGTAGGGGTTGTA-3′ and 5′-TACAACCCCTACAAGAAGTACTCTGAC-3′. TATA-binding protein (TBP) was used as an endogenous control (Hs99999910_m1 and Mm00446973_m1 for human and mouse TBP respectively). Each sample was amplified in duplicate.

### Western-blot analysis

Whole-cell extracts were obtained from VSMC as described^42^. In some experiments, supernatants from TgLOX or wild-type VSMC concentrated with Amicon Ultra 10 K filter units (Millipore) were used^43^. Whole-cell extracts and concentrated cell supernatants were resolved by SDS-PAGE and transferred to 0.45 μm polyvinylidene difluoride membranes (Immobilon, Millipore). Blots were incubated with antibodies directed against LOX (ab31238, Abcam), LOX-PP (NB110-41568, Novus Biologicals) and PCNA (FL-261, Santa Cruz Biotechnology). Bound antibodies were detected after incubation with a HRP-conjugated goat anti-rabbit IgG and using the SuperSignal West Dura Extended Duration Substrate (Pierce). Equal loading of protein was verified by Ponceau staining and by β-actin (A5441, Sigma) or glyceraldehyde 3-phosphate dehydrogenase (GAPDH; MAB374, Millipore) signal as stated.

### Determination of DNA synthesis

*De novo* DNA synthesis, determined by [6-^3^H]-thymidine incorporation into DNA, was used as an index of mitogenic activity^20,41^. VSMC were seeded in 24 well-plates and grown to subconfluency. Then cells were incubated in serum-deprived medium for 48 h and stimulated with complete medium. [6-^3^H]-thymidine (23 Ci/mmol; Amersham) was added to the cells at a concentration of 0.5 µCi/mL, as described elsewhere. After 24 hours, cultures were washed with PBS, fixed with 95% methanol, treated with 10% TCA at 4 °C and dissolved in 0.3 N NaOH. Aliquots were counted on a β-counter (Beckman Coulter^TM^, LS 6500 Multipurpose Scintillation Counter).

### Statistical Analysis

Results are shown as mean ± standard deviation (unless otherwise stated). Significant differences were analyzed using the Student’s t-test (two-tailed) comparing two groups. One-way ANOVA and the Tukey’s test or two-way ANOVA and Fisher’s LSD were used to determine the statistical significance of the observed differences between the studied groups. When normality failed, we used the Mann–Whitney rank sum test to compare two groups or Kruskal-Wallis and the Dunn’s test when more than two groups were compared. Analysis of data was done using the GraphPad Instat programme (GraphPad Software V2.03). Differences were considered significant at p < 0.05.

The datasets generated during and/or analyzed during the current study are available from the corresponding author on reasonable request.

## Electronic supplementary material

Supplemental Information
