# Supplementary material for: Lysyl oxidase (LOX) limits VSMC proliferation and neointimal thickening through its extracellular enzymatic activity
Source: Sci Rep. 2018 Sep 5;8:13258. doi: 10.1038/s41598-018-31312-w (PMC6125287; doi:10.1038/s41598-018-31312-w)
Supplement: Supplementary file 1 — Supplemental Information [file 41598_2018_31312_MOESM1_ESM.pdf]

SUPPLEMENTARY INFORMATION

Lysyl oxidase (LOX) limits VSMC proliferation and neointimal thickening through its extracellular enzymatic activity

Saray Varona, Mar Orriols, María Galán, Anna Guadall, Laia Cañes, Silvia Aguiló, Marc Sirvent, José Martínez-González, Cristina Rodríguez

Supplementary Figure S1

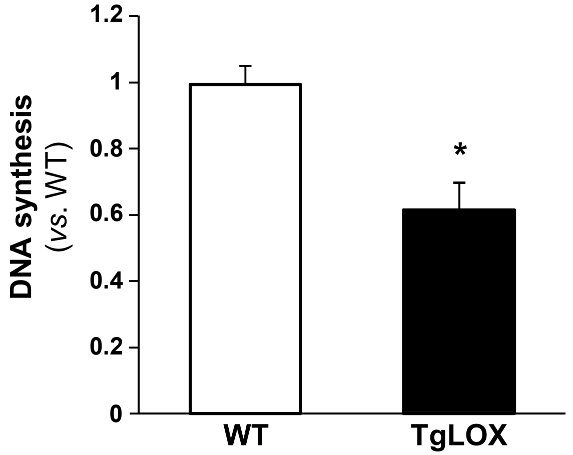

Supplementary Fig. S1. LOX transgenesis reduces VSMC proliferative rates. A. Aortic VSMC isolated from wild type mice (WT; white bars) or TgLOX mice (black bars) were serum-starved for 24 h and then stimulated with 20% FCS. Cell proliferation was analyzed by the [^{3}H]-thymidine incorporation method. Results are represented as mean ± SD. *P<0.01 *vs.* WT cells (Mann–Whitney test; n=5).

Supplementary Figure S2

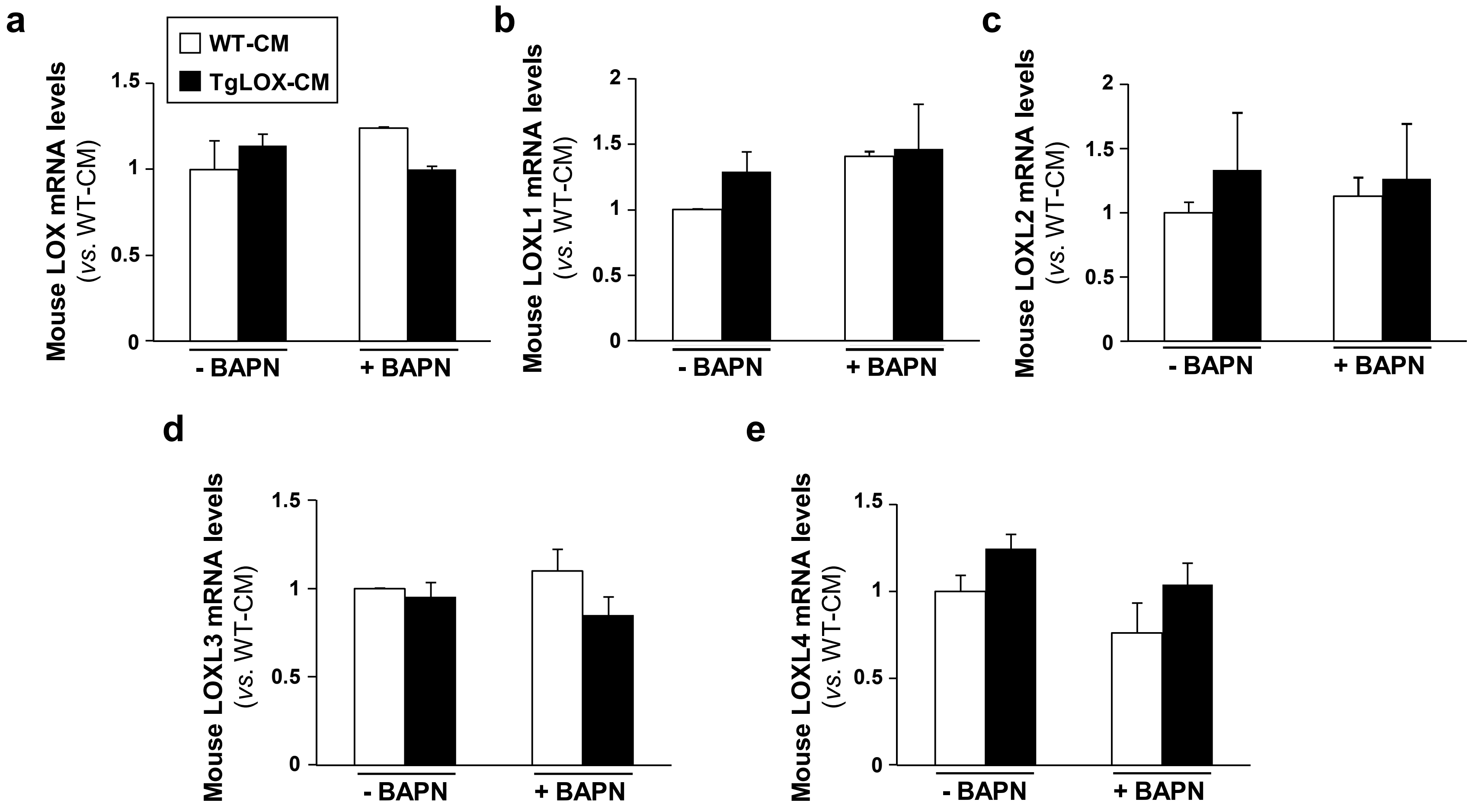

Supplementary Fig. S2. BAPN did not alter the endogenous expression of LOX and LOXL isoenzymes. Aortic VSMC isolated from wild type mice (WT) were serum-starved for 24 h and then exposed to conditioned media from transgenic VSMC (TgLOX-CM; black bars) or wild-type cells (WT-CM; white bars) treated or not with BAPN. LOX **(a)** and LOXLs **(b-e)** mRNA levels were analyzed by real-time PCR. Results are represented as mean ± SD (Two-way ANOVA; n=4).

Supplementary Figure S3

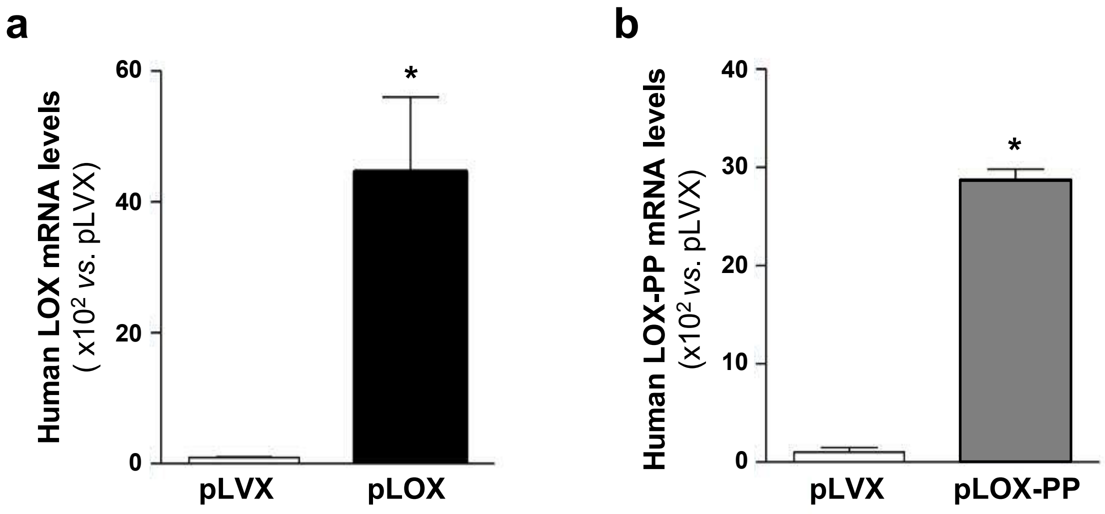

Supplementary Fig. S3. Over-expression of LOX and LOX-PP in VSMC by lentiviral transduction. Human VSMC were transduced with the control lentiviral vector pLVX, pLVX/LOX (pLOX) or pLVX/LOX-PP (pLOX-PP). LOX **(a)** and LOX-PP **(b)** over-expression was verified by real-time PCR. Results are represented as mean ± SD. *P<0.01 vs. pLVX transduced cells (Mann–Whitney test; n=5).
